# Editorial: GPCRs and Cancer

**DOI:** 10.3389/fgene.2017.00162

**Published:** 2017-10-31

**Authors:** Suzie Chen

**Affiliations:** ^1^Susan Lehman Cullman Laboratory for Cancer Research, Ernest Marion School of Pharmacy, Rutgers University, Piscataway, NJ, United States; ^2^Rutgers Cancer Institute of New Jersey, New Brunswick, NJ, United States

**Keywords:** PCR, cancer, excess ligand, targets for treatment, genetic predisposition

In this special e-book “GPCRs and Cancer” there are a collection of comprehensive reviews describing the involvement of GPCRs in cancer. Although the description of the first oncogenic GPCR was over 30 years ago the concept is still relatively foreign to most people. G-protein coupled receptors (GPCRs) comprise a diverse and large group of receptors found in most eukaryotic organisms. There are a variety of ligands which bind and stimulate the receptor including neurotransmitters, chemokines, biogenic amines, peptide and non-peptide hormones, prostanoids, growth factors, nucleosides, lipids, and odorant molecules. Stimulation of the receptor is mediated through the activation of heterotrimeric G proteins. It has become clear within the last few years that GPCRs could function as an oncogene with the identification of the transforming function of *MAS* in 1986 (Young et al., 1986). Unlike most oncogenes identified at that time, the oncogenic activity of *MAS* was not conferred by genetic mutations. Additional studies revealed that GPCRs possess transforming activity by either elevated levels of circulating ligand/agonist or aberrant expression. There are GPCRs with transforming activity by mutation(s) in critical residues without ligand or agonist binding to the receptor (Knox et al., 1969; Hamm, 1998; Li et al., 2005; Lundstrom, 2006; Lee et al., 2008; Lappano and Maggiolini, 2011). Furthermore, the expression level of GPCRs is not as essential in oncogenesis as the receptor simply being expressed. The following is a summary of the reviews from each contributor for this special issue of “GPCRs and Cancer.”

In the first article in this e-book, Swope and Abdel-Malek reviewed the critical roles of two GPCRs, melanocortin 1 receptor (MC1R) and the endothelin B receptor (EDNBR) in melanocytes, specifically their responses to UV-irradiation. Molecular cloning of the MC1R gene and subsequent population studies revealed the highly polymorphic nature of MC1R, particularly some of its variants are non-functional receptors, which are associated with red hair, fair skin, and poor tanning ability phenotypes that have increased melanoma risk (Abdel-Malek et al., 1999, 2000). Normally, functional MC1R when activated by its agonist α-melanocyte-stimulating hormone stimulates eumelanin synthesis and initiates signaling cascades of antioxidant that regulate growth, differentiation, proliferation, apoptosis, DNA repair, and survival. While activation of EDNBR by endothelin-1 reduces the induction of UV-induced DNA photoproducts, in parallel it promotes the repair of such DNA photoproducts (Abdel-Malek et al., 2014).

In the next article, Horrell et al. reviewed structure and function of MC1R and the molecular mechanisms of MC1R signaling in the nucleotide excision repair (NER) pathway. This group demonstrated earlier that MC1R, a Gs-coupled receptor, mediates its signaling through cAMP and protein kinase A (PKA) (Dorsam and Gutkind, 2007). Upon activation, MC1R promotes PKA-mediated phosphorylation of ataxia telangiectasia and rad3-related (ATR) protein at serine residue 435. This results in enhanced NER via recruitment of NER factor XPA and together with ATR-pS435, localizes to sites of UV-induced photo-damage (Jarrett et al., 2014). In the absence of PKA-mediated phosphorylation of ATR at serine 435, there is no enhancement of NER, suggesting MC1R mediates genomic stability through this specific phosphorylation of ATR (Jarrett et al., 2015).

In the third article of this e-book, Rosero et al. described another GPCR, protease-activated receptor (PAR). Unlike other GPCRs where activation occurs via binding of a ligand, PAR activation is mediated through proteolytic cleavage of the N-terminal domain of the receptor by a serine protease. Such cleavage leads to irreversible conformational changes and establishes a new amino terminus that acts as a tethered ligand for activation of the receptor (Vu et al., 1991). There are four types of PARs, PAR1-4. PAR-1 is expressed in many cell types including platelets, endothelial cells, fibroblasts, neurons, T-cells, smooth muscle cells, astrocytes, and others (Macfarlane et al., 2001). It is also detected in a wide variety of cancer cells such as colon, melanoma, prostate and breast (Jin et al., 2016). When stimulated, the PAR family activates numerous intracellular signaling pathways including the well-known MAPK and PI3K pathways that participate in critical cellular processes such as cell proliferation, and cell survival. PAR-1 is involved in the interactions between tumor cells and their microenvironment including cell adhesion, angiogenesis, invasion, and metastasis (Braeuer et al., 2011; Zigler et al., 2011). Targeting PAR-1 with silencing RNA led to reduced tumor cell growth and metastasis (Villares et al., 2008), further suggesting PAR and melanomagenesis.

In the next article by Urtatiz and Van Raamsdonk described the involvement of two specific heterotrimeric G-protein α subunits, GNAQ and GNA11 in melanoma. Frequent oncogenic mutations have been described in these subunits in mouse mutants with a dark dermis from an N-ethyl-N-nitrosourea mutagenesis screen of mice (Hrabe de Angelis et al., 2000; Fitch et al., 2003; Van Raamsdonk et al., 2004). These mutations increased the number of melanocytes in the dermis throughout the life of the mice but did not induce tumor formation. In humans, mutations in GNAQ and GNA11 occur at two hotspots at glutamine 209 and arginine 183. Melanocytic lesions affected by these mutations include development of blue nevi, uveal nevi, uveal melanomas, and leptomeningeal melanocytomas (Van Raamsdonk et al., 2009). Subsequent studies suggest that the developmental lineage of a melanocyte determines if constitutive activation of GNAQ and GNA11 may be oncogenic (Huang et al., 2015).

Finally, in this e-book, Isola and Chen describe the possible involvement of exosomes in tumor metastases. The dissemination of primary tumor cells to distant vital tissues is a major life-threatening complication and the major cause of death in most cancer types. Various host-tumor interactions determine the formation of a secondary metastatic tumor. What determines which distal organ(s) the tumor cells will eventually reside in remains largely unknown. Exosomes are naturally occurring small membrane enclosed microvesicles generated by normal and tumor cells and released by different cell types but more so from tumor cells into the circulation (Baj-Krzyworzeka et al., 2006; Iero et al., 2008). Several reports have proposed that exosomes have the ability to educate bone progenitor cells to become receptive and support tumor cell growth and metastasis (Peinado et al., 2012). Possible involvement of metabotropic glutamate receptor (GRM1), a GPCR that was shown previously to participate in human melanoma (Yu et al., 2014), in exosomal production and/or release was being investigated. Exosomes could be isolated from blood, thus the potential of using this relatively non-invasive means to evaluate outcomes to treatment and provide reliable information for patients' benefit is a possible and practical goal.

We now know that cancer is a genetically heterogeneous disease contributed by mis-regulated expression of many genes and the interactions between them. Numerous GPCRs have been key players in the development and progression of tumors (Bar-Shavit et al., 2016). Emerging evidence in GPCR biology points to the regulation of the receptors by many agonists and antagonists, and these are the most sought-after targets for the treatment of various human diseases. A better grasp on the molecular mechanisms of GPCRs in tumorigenesis will contribute to the development of GPCR therapeutic approaches for the treatment of human cancers.

## Author contributions

The author confirms being the sole contributor of this work and approved it for publication.

### Conflict of interest statement

The author declares that the research was conducted in the absence of any commercial or financial relationships that could be construed as a potential conflict of interest. The reviewer JT and handling Editor declared their shared affiliation.
